# Role of conscious awareness and Big Five in predicting the digital addiction

**DOI:** 10.3389/fpubh.2024.1449847

**Published:** 2024-12-06

**Authors:** Yıldız Erzincanli, Fatma Geçikli

**Affiliations:** ^1^Aşkale Vocational High School, Department of Office Management and Executive Assistance, Atatürk University, Erzurum, Türkiye; ^2^Department of Public Relations and Advertising, Public Relations, Faculty of Communication, Atatürk University, Erzurum, Türkiye

**Keywords:** behavioral addiction, digital addiction, personality, Big Five, conscious awareness (mindfulness)

## Abstract

**Introduction:**

Digital addiction is discussed in the literature as a type of addiction that negatively affects the personal, social, and societal lives of individuals. Digital addiction is a behavioral addiction that occurs as a result of the problematic and unconscious use of digital tools with features such as compulsive, excessive, impulsivity, and includes human-machine interaction. What is meant to be expressed with conscious awareness is to accept the facts and experiences as they are without judgment, and to be aware of them. With awareness, it is aimed to contribute to individuals to evaluate their past experiences more objectively, to get rid of automatic behavioral and emotional patterns, and to show more moderate reactions by avoiding aggressive reactions. In this context, this study examines the Big Five and Mindfulness as predictive variables for digital addiction, focusing on them as a cause and a solution variable that can be used in interventions for digital addiction. Given its prevalence and negative effects, it is important to identify and analyze the relationship patterns between digital addiction and related variables in order to define and resolve the problem. In this context, the present study aims to determine the levels of conscious awareness and digital addiction among university students and to examine the predictive effect of conscious awareness levels and Big Five variables on predicting digital addiction behaviors.

**Methods:**

The present study is designed to employ the survey method. Data were collected from 1,664 university students selected by using the convenience sampling method, and the data obtained were analyzed by using descriptive statistics and structural equation modeling. The SPSS 25 and LISREL 8.8 software packages were used in the analysis process.

**Results and discussion:**

The results achieved in this study revealed that university students have a moderate level of conscious awareness and that the overall level of digital addiction is generally moderate. The results achieved from the structural equation modeling for the measurement model constructed for the relationships between variables confirmed the validity of the proposed model. It was determined that the model had a good fit with the latent variables, which represent the indicator variables, and also other latent variables. In conclusion, it was found that Big Five and the level of conscious awareness have a reducing effect on digital addiction and serve a protective function against this negativity among university students.

## 1 Introduction

Information technologies are rapidly developing and diversifying in today’s world (1, 2). Digital addiction is one of the problems encountered due to the effect of technology on lifestyles (3–5). Digital addiction is a behavioral addiction that arises from the problematic and unconscious use of digital tools, which is characterized by features such as compulsion, excessiveness, and impulsivity, and incorporates the human-machine interaction (6, 7). Although digital addiction is the main concept, there also are studies on subtypes such as gaming addiction, internet addiction, and social media addiction in the literature. Prevalence studies in the general population indicate a prevalence rate of around 25% for digital addiction and/or its subtypes (8). Digital addiction encompasses many aspects of compulsive digital device use, such as smartphone addiction (including non-mobile phobia: nomophobia), problematic use of the internet and social media, cyberchondria, cybersex addiction, etc., under the umbrella term PUI (9). Given that a substantial section of the world’s population may be impacted by online activities and compulsive internet use, the World Health Organization (WHO) formally acknowledged digital device addiction as a widespread problem in 2020 (5). The findings of a meta-analysis carried out in 2022 indicate that the prevalence of digital addiction has risen during the previous two decades (8). Furthermore, 15% of youth may have a digital addiction, according to a poll conducted by the American Medical Association. Digital addiction does not necessarily involve internet usage; it includes not only addiction to online activities but also offline activities that use digital devices, such as offline gaming addiction (10).

Digital addiction is an important concept that is being researched worldwide and has been a subject of various studies in recent years. Especially among young individuals, the problem of digital addiction is becoming increasingly evident (11). Digital addiction can cause significant harm to individuals’ psychological, physical, and mental development. It has been determined that digital addiction harms individuals’ healthy development by causing headaches and chronic (low) back pain, decreased visual acuity, delays in physical development, and weakened social interaction. Nowadays, studies are being conducted on the factors and mechanisms affecting digital addiction. The misuse of digital tools can harm both physical and mental health in individuals, leading to serious problems not only for individuals but also for families and communities (12–15).

Similar to substance abuse, digital addiction is classified among behavioral addictions and has characteristics such as withdrawal, relapse, and cognitive preoccupation (16–18). Two fundamental similarities between digital addiction and behavioral addictions are the presence of a medium (the internet) and the role of personality traits (19–21). Personality traits refer to the fundamental characteristics influencing an individual’s behavior (22, 23). Additionally, McMartin (24) described personality as a consistent system with motivational, emotional, and cognitive integrity, distinguished by unique reactions and behaviors exhibited by an individual throughout their life. Studies highlight the importance of personality traits in the diagnosis and treatment of addictive behavior, considering individual characteristics as fundamental factors in the process leading to digital addiction (25). Different individuals with varying personality traits exhibit different usage patterns and addiction reasons for communication tools within social life. In other words, personality creates specific conditions for addiction (26, 27).

There are many personality traits identified in individuals as a result of the researches, the “5 Factor Personality Model” has been a very useful discrimination method in digital addiction studies and has brought a current functional perspective to the concept of personality. Personality creates special conditions for addiction (28). According to the five dimensions of personality in the five factor theory, introverts have low levels of success and ability to establish relationships in interpersonal environments, since they are not socially compatible and are quiet and shy. For this reason, since they use social opportunities less in their daily lives, they turn to virtual relationships in the technology environment in order not to be alone, and because they have an important life in the virtual environment, they cannot use technology in a controlled manner and increase their addiction level. Introverted, emotionally unstable, maladaptive individuals are more likely to use technology to become addicted than individuals with openness and responsibility. People with high responsibility are organized and disciplined. Extraverts are curious. For this reason, they tend to virtual environments and virtual relationships less in their social lives than the other five factor dimensions (29).

Examining studies on digital addiction and personality, it can be seen that there are significant correlations between them since there are different types of digital addiction and the factors underlying these different addiction types are common (30). Studies on the profiling of different technology addiction behaviors by making use of personality traits reported remarkable results (20, 31). In their study on undergraduate students, Erdem and Uzun found that the five major personality traits significantly predicted smartphone addiction (32). Hawi and Samaha, in their study on commonalities and differences in personality traits related to internet and social media addiction, determined that personality predicts both internet and social media addiction (20). Parmaksız examined the mediating role of personality traits in the relationship between university students’ digital addiction behaviors and academic self-efficacy, finding that personality traits fully mediated the relationship between digital addiction and academic self-efficacy (25).

As a result, it is considered that an individual’s personality traits play a significant role in the type, causes, and level of addiction, and they can also be made use of to manage the addiction tendencies (33, 34). Therefore, it is very important to examine the relationship between digital addiction and personality in terms of multiple predictors.

Another variable addressed in the study concerning digital addiction is conscious awareness (mindfulness). As defined by Kabat-Zinn, mindfulness is ‘*intentionally paying attention to the present moment without judgment’* (35). However, Siegel et al. stated that mindfulness involves accepting experiences in the present without judgment (36). Mindfulness contributes to individuals evaluating their past experiences more objectively, disengaging from automatic behavioral and emotional patterns, and exhibiting milder responses (37, 38). In a study examining smartphone addiction and health outcomes of young and older adult consumers in relation to their mindfulness characteristics, Kim and Milne revealed that unconscious behaviors are caused by environmental stimuli leading smartphone users into automatic habits and engagement with smartphone use and they also emphasized the importance of increasing awareness and promoting mindfulness education as a solution (39). Similar studies on smartphone addiction indicated that mindfulness has a significant effect on improving smartphone addiction (40) and that it regulates associated distressing emotional states (41).

Furthermore, mindfulness is an effective and solution-focused approach in combating digital addiction (42–44). Applications and interventions of mindfulness were observed to be effective in various types of addiction, such as substance addiction, social media addiction, and internet addiction (45, 46). By making use of mindfulness education and practices, addicted individuals can be treated and reductions can be achieved in addiction levels since they have an awareness of their emotions, thoughts, behaviors, and reactions (40, 47, 48). Previous studies suggested that mindfulness should be examined not only as a limited intervention to prevent addiction relapse but also as a long-term and sustained health behavior contributing to addiction recovery (49, 50). In conclusion, mindfulness can encourage individuals to focus more objectively on the present moment, activate their coping abilities with the present time, and strengthen self-regulation skills, which enable individuals to control their tendencies toward digital addiction. Therefore, it is very important to examine the relationship between digital addiction and mindfulness in terms of multiple predictors.

Individuals differ in terms of their personality traits. Whether an individual is emotionally balanced or not and also their extroverted or introverted behaviors can affect the level of conscious awareness (51–53). Within this context, Brown and Ryne claimed that there is a significant relationship between conscious awareness and personality traits (37). Examining the relevant literature, significant relationships between conscious awareness and personality traits were determined (54, 55). In their meta-analysis, Hanley and Garland found significant relationships between mindfulness and personality traits in studies focusing on the relationship between mindfulness and personality traits (54). Westbrook also reported that personality traits significantly predict conscious awareness (56). Simultaneous evaluation of personality in relation to awareness is important for determining unique relationships by controlling mutual relationships among personality traits. In light of this perspective, examining the relationship between conscious awareness and personality in terms of multiple predictors is very important.

Parallel to developments in the field of psychology, interest in mindfulness meditation training in the fight against addiction has increased. Standardized mindfulness training programs initially focused on reducing emotional distress, and indeed, mindfulness-based interventions (MBIs) for psychiatric disorders and symptoms have been proven effective and comparable to other active treatments through meta-analysis (46). MBIs have been found to provide significant clinical benefits for a range of addictive conditions, including alcohol, cocaine, nicotine, and technology.

In light of the findings resulting from both intervention and descriptive studies on digital addiction, a model was constructed in this study to determine the multiple predictive relationships between personality variables and mindfulness. Examining the relevant literature, it was noted that the number of studies examining the predictive role of mindfulness and personality in predicting the level of digital addiction is limited. Therefore, this study aims to provide novel insights. It is important to raise awareness regarding digital addiction and understand variables that affect and resolve digital addiction. Since personality is associated with a variety of behaviors leading to digital addiction, it might be discussed if mindfulness has an effect on digital addiction through personality. It is thought that personality traits might have significant effects on the relationship between digital addiction and mindfulness. In digital/technological addictions, as in other addictions, if early intervention is not made, many negative situations such as communication problems, decreased work performance, failure, and failure to fulfill responsibilities may occur in the individual. In this context, researchers need to prioritize studies on how to control technology behaviors that cause addiction in individuals.

Thus, this study aims to determine whether personality and mindfulness play a meaningful role in combating digital addiction.

In this study, the focus is on a cause variable for digital addiction and a solution variable that can be used in interventions for digital addiction. Therefore, in this study involving young adults, the relationship mechanisms between the factors affecting digital addiction and providing solutions have been examined to provide a theoretical basis and practical support for preventing individuals’ digital addiction and protecting their physical and mental health.

As a result, the aim of this study was to determine the predictive relationships between personality, conscious awareness and digital addiction variables. In this context, a model was created. In the created model, a general answer was sought to the question of whether the personality and conscious awareness levels of university students have a significant effect on digital addiction.

For this purpose, the following hypotheses were tested:

*H1*: There is a significant relationship between personality and conscious awareness, and personality serves as a meaningful predictor of conscious awareness.

*H2*: There is a significant relationship between personality and digital addiction, and personality acts as a predictor of digital addiction.

*H3*: Personality and conscious awareness jointly have a predictive effect on digital addiction.

## 2 Method

### 2.1 Participants and demographic characteristics

Because of its higher prevalence among adolescents and young adults (25), problematic digital technology use is an important problem since it directly affects all students. The sample of this study consists of 1,664 voluntary students selected from a university in Turkey by using the convenience sampling method. Of the participants, 67.1% are female, and 32.9% are male. Sixty-four percent of the participants are within the age range of 19–29, 23.4% within the age range of 30–39, 9.5% within the age range of 40–49, and 3.1% within the age range of 50–59. The demographic characteristics of the participants are presented in Table 1.

**Table 1:** Demographic characteristics.

Variables		F	%
Gender	Female	1,084	67.1
	Male	532	32.9
Marital status	Married	599	37.1
	Single	938	58.0
	Other	79	4.9
Employment status	Employed	890	55.1
	Unemployed	726	44.9
Residing in	City	1,145	70.9
	District	328	20.3
	Village	143	8.8
GPA	1.99 and less	232	14.4
	2.00–2.99	831	51.4
	3 and higher	553	34.2
Average hours of using digital tools per day	1–2 h	786	48.6
	3–4 h	565	35.0
	5–6 h	265	16.4
Age	19–29 years	1,027	64.0
	30–39 years	327	23.4
	40–49 years	153	9.5
	50–59 years	49	3.1
Mean of age	32.78	Standard deviation for age: 84.70	

### 2.2 Data collection tools

#### 2.2.1 Personal information form

The survey includes 20 items related to the demographic and other personal characteristics of the students participating in this study. These items were created by the researcher. They address variables of university students such as gender, age, program of study, class level, academic achievements, marital status, employment status, income level, social media restrictions, place of residence, family types, and time spent on social media.

#### 2.2.2 Digital dependency scale

The scale developed by Kesici and Tunç was utilized to measure students’ levels of digital addiction. Comprising five sub-dimensions, namely “*Excessive Use*,” “*Relapse*,” “*Disruption of Life Flow*,” “*Mood*,” and “*Inability to Quit*,” this scale consists of 19 items rated on a 5-point Likert scale (57). In this study, the structural validity of the Digital Addiction Scale was examined by using exploratory factor analysis, and it was determined that the 5-factor structure explained 49% of the variance. Moreover, the reliability coefficient of the scale was found to be 0.89 for the 5 dimensions and 19 items. The internal consistency coefficient of the scale, which was determined to be 0.85 in the adaptation study, was calculated to be 0.86 in the present study.

#### 2.2.3 Five-factor personality inventory

To identify students’ personality types, a ten-item Five-Factor Personality Inventory, consisting of five sub-dimensions with two items each, was employed. This scale was developed by Rammstedt and John and adapted to Turkish culture by Horzum et al. (58). The original dimensions of the scale, namely “Extraversion,” “Agreeableness,” “Conscientiousness,” “Neuroticism,” and “Openness to Experience,” were retained. The reliability of the scale was determined by achieving internal consistency and composite reliability values exceeding 0.70, indicating high reliability (58). The internal consistency coefficient of the adapted scale was established as 0.88 in the adaptation study, while in the present research, it was measured at 0.84.

#### 2.2.4 Mindful attention awareness scale

Developed by Brown and Ryan (37) in order to assess the conscious awareness levels of students, the Mindful Attention Awareness Scale was adapted to Turkish by Özyeşil et al. (59) in a study involving a cohort of university students. The scale was found to have a high reliability, with a Cronbach’s Alpha internal consistency coefficient of 0.80 and a test–retest correlation of 0.86 determined in the adaptation study (49). The internal consistency coefficient of the scale, which was found to be 0.80 during the adaptation process, was calculated to be 0.82 in the present study. The data obtained suggest that all the scales used in this study had sufficient validity and reliability, which makes them suitable for application throughout the research process (59).

### 2.3 Procedure

This study was designed by using the relational scanning model. Accordingly, participants were provided with necessary explanations regarding the purpose of the study, the anonymous use of the obtained data, and the principle of voluntary participation in this study in order. Due to the pandemic, the data for the study were collected in 2022 by distributing a survey link on the university webpage via Google Forms. The link was visible exclusively to students accessing the university page with their user credentials.

### 2.4 Data analysis

In this study, the homogeneity of the data was initially tested, and relationships between variables were examined. There was no missing part in the data of any participant involved in the present study. The data were transferred from Excel to SPSS and then subjected to extreme value analysis. It was determined that 48 participants out of the total 1,664 in the dataset had extreme values, which were then excluded from the dataset. The study universe consists of students studying at Atatürk University, whereas the sample consists of those enrolled in the Open Education Faculty during the 2020–2021 academic year. To represent the study sample within the context of this study, students registered in the associate and undergraduate programs of Atatürk University’s Open Education Faculty during the 2020–2021 academic year were included. The total number of students at Atatürk University Open Education Faculty is 496,887. Considering the sampling table prepared by (60), with a sampling error of 0.03 and a *p*-value of 0.5, a sample size of 1,066 is considered sufficient. Accordingly, data collection was completed with 1,166 associate degree students and 450 undergraduate students from the open education faculty. If a researcher wants to reduce the error in his analysis, he should increase the sample size (61). In this study, the sample size was increased to reduce the error in the analysis and a total of 1,664 data points were obtained.

In the analysis of the data, validity and reliability analyses were first conducted to test the measurement properties of the scales used in the research. In the validity and reliability phase, it was determined that there were no problems with the scale items. Subsequently, exploratory factor analysis (EFA) was applied to the factor structure of the scales, followed by confirmatory factor analysis (CFA), to determine the validity and reliability levels of the scales. These levels are provided in the scales section. During the research process, the relationship patterns between personality structure, level of conscious awareness, and digital addiction were examined using structural equation modeling. Structural Equation Models (SEM) are a statistical modeling technique. It reveals the cause-and-effect relationship between measured and unmeasured variables in a study.

According to the standard normal distribution, the 95% confidence interval requires the z value to be within the range of ±3. For this reason, the data of 48 participants who were determined not to be within this range in the calculation of the z values of the study were removed from the data set. Apart from the extreme value analysis, the skewness kurtosis values were examined and the data were shown to be normally distributed with histograms and scatter plots, and therefore the analyses were continued with parametric tests (62).

Following the completion of extreme value analysis, the arithmetic mean, skewness, and kurtosis values were calculated in order to determine whether the normality assumption for parametric tests was met. The calculations indicated that the data has a normal distribution, as shown in Table 2.

**Table 2:** Descriptive statistics.

Variables	*N*	*Xˉ*	*SS*	*Min.*	*Max.*	*Skewness*	Kurtosis*
Conscious awareness	1,616	60.84	11.51	27	90	−0.080	−0.355
Personality scale	1,616	31.70	3.79	17	44	−0.191	0.199
Extroversion	1,616	7.18	2.06	2	10	−0.438	−0.503
Agreeableness	1,616	3.79	1.42	0	8	0.515	−0.281
Self-control	1,616	8.12	1.60	4	10	−0.585	−0.512
Neuroticism	1,616	5.62	1.96	2	10	0.170	−0.571
Openness to experience	1,616	6.99	1.86	2	10	−0.151	−0.522
Digital addiction	1,616	45.78	12.39	19	84	0.192	−0.287
Excessive use	1,616	11.25	3.82	5	22	0.387	−0.389
Relapse	1,616	6.84	3.04	3	15	0.615	−0.358
Disruption of life flow	1,616	8.42	3.45	4	18	0.638	−0.280
Mood	1,616	8.98	3.11	4	18	0.417	−0.380
Inability to quit	1,616	10.30	2.83	3	15	−0.548	−0.038

Kurtosis* = Kurtosis-3.

**Figure 1:**
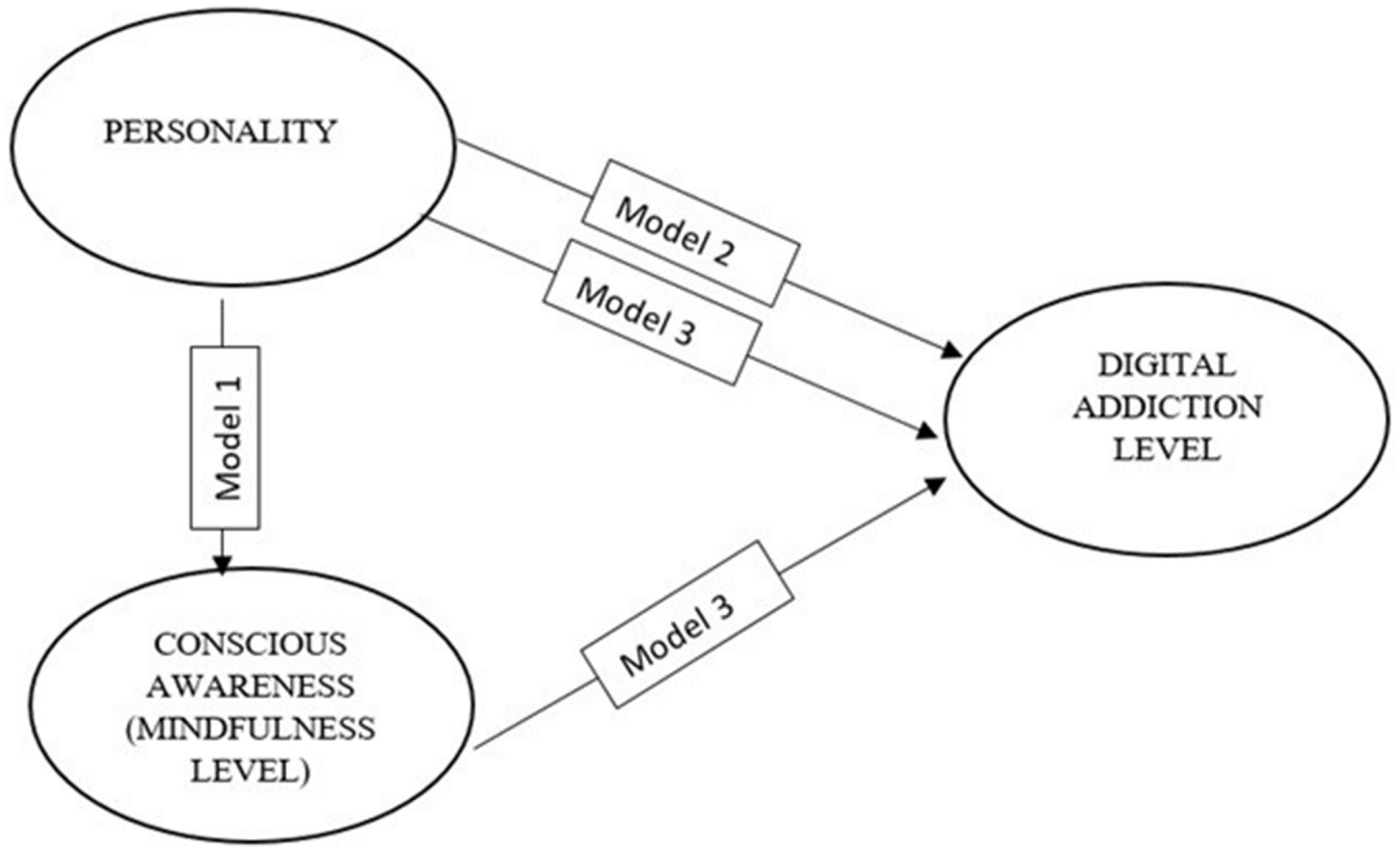
Structural model of the study.

Secondly, Lisrell 8.8 was used to test the structural model presented in Figure 1. The research process examined patterns of relationships between personality structure, conscious awareness level, and digital addiction using structural equation modeling. Before conducting the structural equation modeling to examine the relationship patterns between variables and possible mediation roles, it was investigated whether the variables have statistically significant relationships with each other. The obtained results are presented in Table 3. As presented in Table 3, it has been found that all the variables/sub-dimensions to be included in the structural equation modeling show statistically significant relationships with each other.

**Table 3:** Correlation analysis results.

	1	2	3	4	5	6	7	8	9	10	11
1	1	0.275**	−0.298**	0.314**	−0.354**	0.196**	−0.362**	−0.361**	−0.375**	−0.353**	−0.213**
2		1	−0.131**	0.356**	−0.353**	0.249**	−0.156**	−0.198**	−0.258**	−0.176**	−0.106**
3			1	−0.168**	0.217**	−0.117**	0.243**	0.204**	0.212**	0.229**	0.132**
4				1	−0.294**	0.247**	−0.251**	−0.296**	−0.358**	−0.255**	−0.161**
5					1	−0.234**	0.262**	0.304**	0.268**	0.259**	0.249**
6						1	−0.125**	−0.174**	−0.179**	−0.105**	−0.074**
7							1	0.606**	0.472**	0.480**	0.443**
8								1	0.622**	0.495**	0.356**
9									1	0.494**	0.234**
10										1	0.452**
11											1

1: Mindfulness; 2: Extraversion; 3: Agreeableness; 4: Self-control; 5: Neuroticism; 6: Openness to experience; 7: Overuse; 8: Relapse; 9: Impeding the flow of life; 10: Mood; 11: Inability to let go.

These are subscales of the 2–6 personality scale. It is a subscale of digital addiction between 7 and 11.

Patterns of relationships constructed among variables were analyzed using latent variables, and prior to testing these models, a confirmatory measurement model was constructed to examine fit values among latent variables. In the confirmatory measurement model, three different latent variables, representing personality, digital addiction, and conscious awareness, and the latent variables representing these indicator variables were included in the model. In this context, 10 different indicator variables for personality, 5 different indicator variables for digital addiction, and 15 different indicator variables for conscious awareness were included in the model. Fit indices for the measurement model (*χ*^2^/sd = 2.30; RMSEA: 0.062, RMR: 0.053, SRMR: 0.066, NFI: 0.97 CFI: 0.98, GFI: 0.96) indicate that the constructed model is confirmed, and all latent variables have a good level of fit with the indicator variables they represent and with other latent variables. With the confirmation of the measurement model, the following models were tested in line with the research objectives, and the hypotheses were confirmed.

Model 1 examines the predictive relationships between personality structure and conscious mindfulness.

Model 2 examines the predictive relationships between personality structure and digital addiction.

Model 3 examines the combined predictive effect of personality structure and conscious mindfulness on digital addiction.

## 3 Results

In this study, conscious awareness, personality, and digital addiction scales were administered to participants. Descriptive statistics, including mean, standard deviation, maximum and minimum scores, kurtosis, and skewness values, for the applied conscious awareness, personality, and digital addiction scales are presented in Table 2. The conscious awareness scale is unidimensional, and the arithmetic mean (Xˉ) for the scores obtained from participants is 60.84, with a standard deviation of 11.51. The personality scale, the second scale used in the research process, has an arithmetic mean total score of *Xˉ* = 31.70, with a standard deviation of 3.82. The extraversion subscale has a mean score (Xˉ) of 7.18 with a standard deviation of 2.06, the agreeableness subscale has a mean score (Xˉ) of 3.79 with a standard deviation of 1.42, the self-control subscale has a mean score (Xˉ) of 8.12 with a standard deviation of 1.6, the neuroticism subscale has a mean score (Xˉ) of 5.62 with a standard deviation of ±1.9, and the openness to experience subscale has a mean score (Xˉ) of 6.99 with a standard deviation of ±1.8. For the digital addiction scale, the arithmetic mean total score is *Xˉ* = 45.78, with a standard deviation of 12.37. Subscale mean scores are as follows: excessive use *Xˉ* = 11.25 with a standard deviation of 3.8, relapse *Xˉ* = 6.84 with a standard deviation of 3.0, disruption of life flow *Xˉ* = 8.42 with a standard deviation of 3.4, mood *Xˉ* = 8.98 with a standard deviation of 3.1, and inability to quit *Xˉ* = 10.30 with a standard deviation of 2.8.

**Figure 2:**
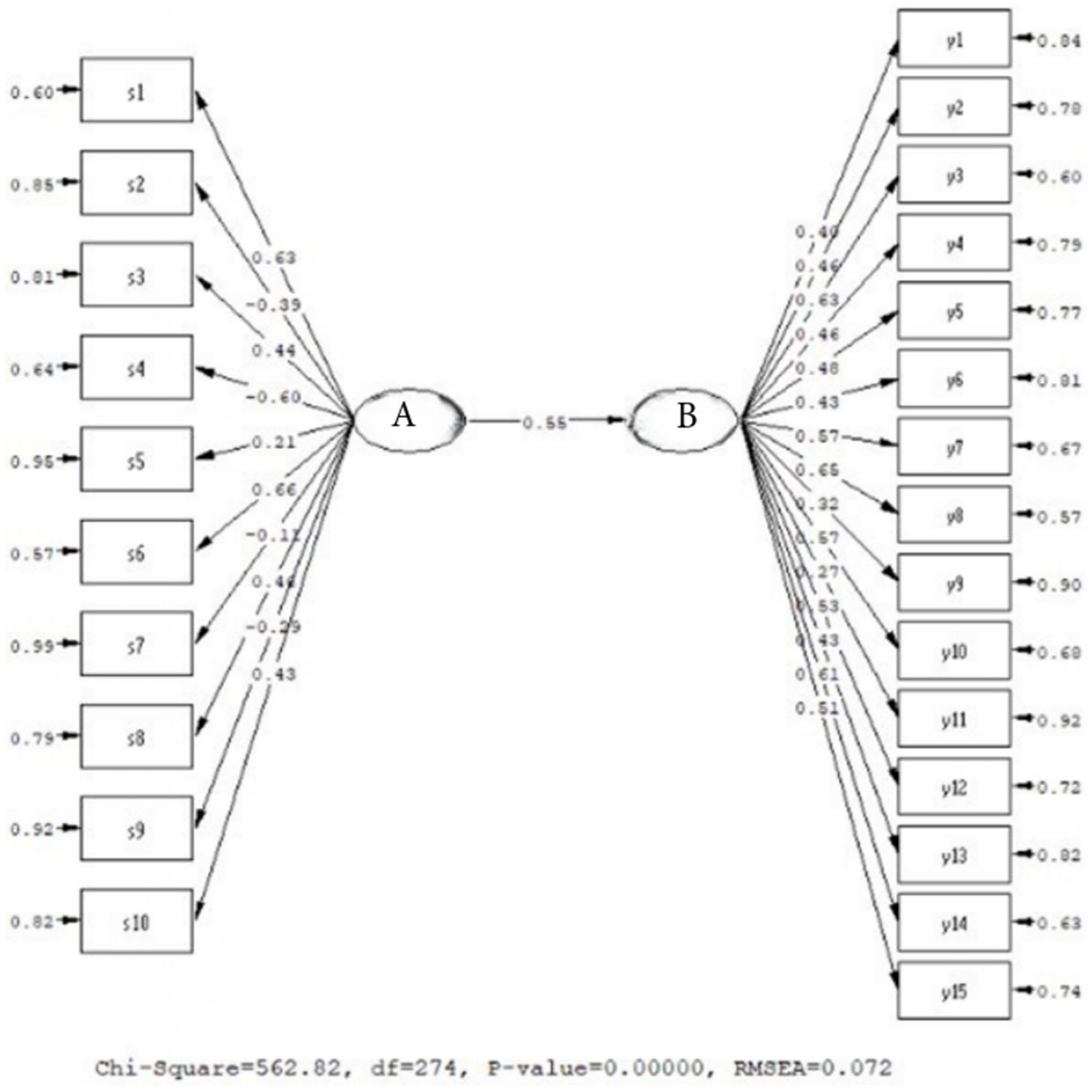
Predictive relationship model for personality and conscious awareness. A: Personality, B: Conscious awareness.

Examining the prediction coefficients presented in Figure 2, it is evident that the model constructed between Personality and Mindfulness is confirmed and it demonstrates a good fit (*χ*^2^/sd = 2.05; CFI = 0.97; TLI = 0.95; RMSEA = 0.072). Accordingly, the prediction coefficients between personality and mindfulness are positively and significantly related (*β* = 0.55, *p* < 0.01), and it can be stated that Personality explains 30% of the variance in Mindfulness. Therefore, the personality structure can be considered a factor that enhances mindfulness in university students. Additionally, we can say that there is a strong relationship between individuals’ personality traits and their levels of conscious awareness, and that personality traits affect the level of conscious awareness.

**Figure 3:**
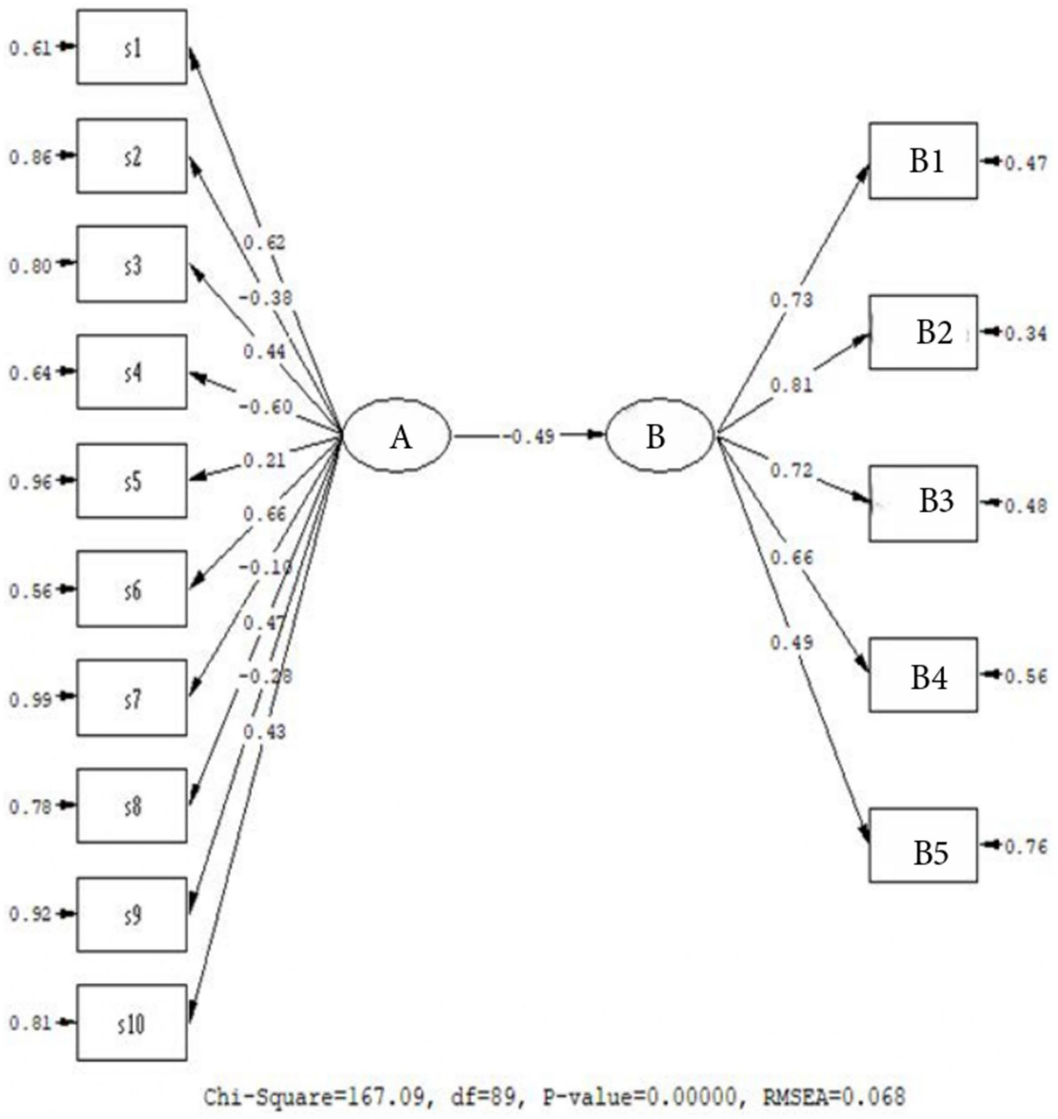
Predictive relationship model for personality and digital addiction. A: Personality, B: Digital addiction, B1: Overuse, B2: Non-restraintB3: Inhibiting the flow of life, B4: Emotional state, B5: Dependence.

Examining Figure 3, it was determined that the model formulated to predict the relationship between Personality and Digital Addiction is confirmed and it exhibits a good fit (*χ*^2^/sd = 1.75; CFI = 0.98; TLI = 0.97; RMSEA = 0.068). Accordingly, the prediction coefficients for the prediction between personality and conscious awareness were found to be negative and significant (*β* = −0.49, *p* < 0.01), and it can be stated thattab Personality explains 25% of the variance in Digital Addiction. Therefore, the personality structure has an inverse relationship with digital addiction in university students, and in this regard, it can be suggested that specific personality traits that have high values might have a function reducing digital addiction. It can also be said that individuals’ personality traits are a determining factor in their levels of digital addiction. We can say that personality traits should be prioritized when examining individuals’ addiction behaviors.

**Figure 4:**
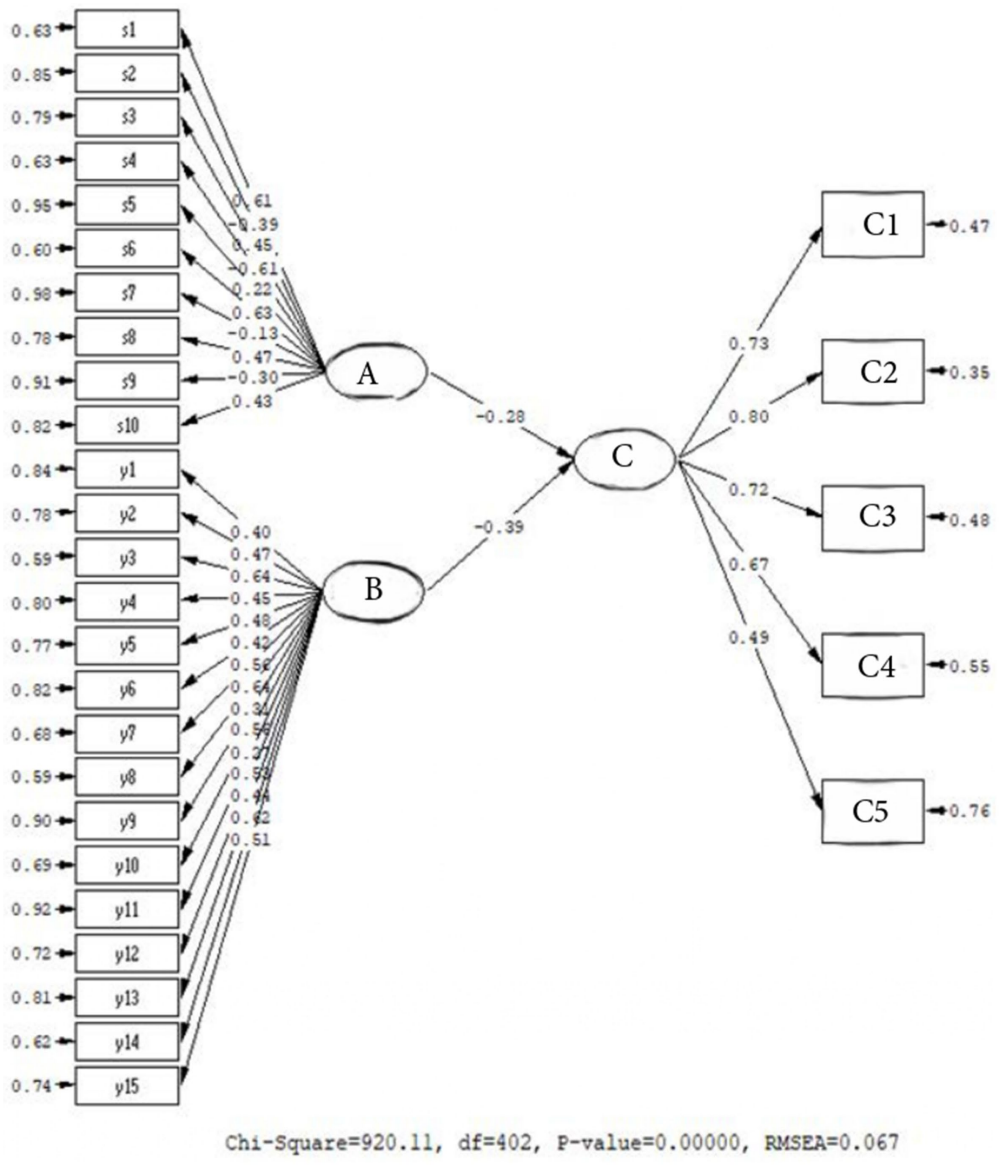
Multiple prediction relationship between personality, conscious awareness, and digital addiction. A: Personality, B: Conscious Awareness, C: Digital AddictionC1: OveruseC2: Non-restraint, C3: Inhibiting the Flow of Life, C4: Emotional State, C5: Dependence.

Figure 4 shows that the model examining the combined predictive effect of personality and conscious awareness on digital addiction is confirmed and it has a good fit (*χ*^2^/sd = 2.28; CFI = 0.98; TLI = 0.98; RMSEA = 0.067). Accordingly, Personality and Conscious awareness jointly strongly predict Digital Addiction. When considering the variables, Personality explains 7% of the variance in digital addiction (*β* = −0.28, *p* < 0.01). Conscious awareness, on the other hand, accounts for 15% of Digital Addiction (*β* = −0.39, *p* < 0.01). The predictive relationship of both variables with Digital Addiction is negative. In this sense, it can be stated that specific personality types and the level of conscious awareness have a reducing effect on digital addiction in university students and serve a protective function against this negativity in youth. Additionally, when examining the digital addiction levels of university students, we can say that both their personality traits and levels of conscious awareness should be taken into account (Table 4).

**Table 4:** Compliance index reference values.

Compliance measure	Good fit	Acceptable adjustment
χ^2^/sd	0 ≤ χ^2^/sd ≤ 2	2 ≤ χ^2^/sd ≤ 3
RMSEA	0 ≤ RMSEA≤0.05	0 ≤ RMSEA≤0.08
RMR	0 ≤ RMR ≤ 0.05	0 ≤ RMR ≤ 0.08
SRMR	0 ≤ SRMR≤0.05	0.05 ≤ SRMR≤0.10
NFI	0.95 ≤ NFI ≤ 1.00	0.90 ≤ NFI ≤ 0.95
CFI	0.97 ≤ CFI ≤ 1.00	0.95 ≤ CFI ≤ 0.97
GFI	0.95 ≤ GFI ≤ 1.00	0.90 ≤ GFI ≤ 0.95

## 4 Discussion

Digital addiction is a psychosocial problem that progressively develops from childhood to adulthood and is associated with various issues (63). Therefore, this study provides important insights in order to mitigate the negative effects of digital addiction in individuals. Digital addiction is a complex issue requiring interdisciplinary research and seeking solutions. Studies carried out worldwide examined the relationship between individuals’ personality traits, digital addiction, and the effect of addiction levels on their physical and mental health, even on their social lives. Previous studies in the literature include those examining the levels of digital addiction and conscious awareness. However, no similar studies predicting and proposing models for the relationship between personality, digital addiction, and conscious awareness have been identified. Consequently, this study proposes and validates a model aimed at reducing digital addiction using structural equation modeling, an advanced statistical technique. Additionally, given that the present study focused on university students (*N* = 1,664), which is a sample group highly affected by the relevant issue, it can be considered a significant and comprehensive contribution to the literature on digital addiction.

In this study, the participants’ levels of digital addiction scores were assessed considering the maximum and minimum scores achievable for each sub-dimension. In this study, as a result of the evaluation of the participants’ digital addiction score levels according to the maximum and minimum scores that can be obtained from each sub-dimension, it was determined that they were in the overuse sub-dimension (*Xˉ* =/min: 5 max: 25 / 11.25), in the relapse sub-dimension (*Xˉ* =/min: 5 max: 15 / 6.84), in the obstruction of the flow of life sub-dimension (*Xˉ* =/min: 5 max: 20 / 8.42), in the mood sub-dimension (*Xˉ* =/min: 5 max: 20 / 8.98), and in the inability to quit sub-dimension (*Xˉ* =/min: 5 max: 15 / 10.30). Studies examining addiction levels in the literature reported both different and similar results. For instance, Aktan (64) found low levels of social media addiction among university students in a study, whereas Arslan (65) reported moderate addiction levels in all sub-dimensions among university students. A study carried out by Ho et al. (66) on social media addiction indicated high addiction levels. It is thought that variations in samples and locations contribute to the diverse outcomes in addiction levels.

Examining the conscious awareness levels as the scores increased, depending on the scale used. The average conscious awareness score for university students in this study was X̄: 60.84, indicating a moderate level of conscious awareness. A study focusing on university students also noted a moderate level of conscious awareness among participants (67). Similarly, in a study carried out by Ramli et al. (68) examining the relationship between stress and conscious awareness among Malaysian undergraduate students, a moderate level of conscious awareness was reported. Consequently, the findings of this study align with the results of numerous studies conducted both in Turkey and other countries, indicating moderate levels of conscious awareness.

(The relationship between Big Five and conscious awareness).

Firstly, in Model 1 constructed within the framework of the structural equation model, Hypothesis 1 was confirmed, which indicates that personality structure is a factor predicting mindfulness among university students. The relationships between mindfulness and personality traits were examined in two meta-analyses (54, 69). In both meta-analyses, significant relationships were found between mindfulness and personality traits. In a study carried out by Karl et al. (70), which investigated the relationships between different aspects of mindfulness and personality traits in adults, it was found that mindfulness generally exerts strong longitudinal effects on personality. They propose pathways for personality development through mindfulness interventions. Haliwa et al. (71), in their study exploring the extent of the relationship between personality traits and mindfulness, noted that the relationships between mindfulness and personality traits are generally consistent across mindfulness measures. In a study with university students, Nam and Akbay (72) concluded that personality traits significantly predicted life satisfaction through mindfulness and resilience variables. Similar findings were reported in various studies (2, 3, 51), highlighting a meaningful relationship between personality and mindfulness. In a meta-analysis study examining the relationship between the five-factor personality theory and affect with conscious awareness, it was concluded that neuroticism, one of the five factors, has a significant negative relationship with conscious awareness and a significant positive relationship with responsibility (66). In Yiğit’s (2021) study, it was determined that there is a positive relationship between mindfulness and Extraversion, Conscientiousness, Agreeableness, and Openness to Experience, and a negative relationship with Emotional Instability. Additionally, when examining the relationship between the sub-dimensions of the five-factor personality scale and levels of conscious awareness within the scope of this study, it was found that the conscious awareness scale has a weak positive relationship with ‘Extraversion’; a moderate positive relationship with ‘Conscientiousness’; a weak positive relationship with ‘Agreeableness’; a very weak positive relationship with ‘Openness to Experience’; and a weak negative relationship with ‘Emotional Instability’ (89). In the study conducted by Haliwa et al. (28) examining the relationship between mindfulness and the Big Five personality traits, it was found that there is an inversely proportional relationship between mindfulness and neuroticism, and a positively significant relationship with extraversion, agreeableness, conscientiousness, and openness. The results of this study, in parallel with the literature, indicate that personality traits influence and support mindfulness among university students. Consequently, it is recommended to consider personality traits while examining mindful behaviors in university students.

(The relationship between Big Five and Digital addiction).

Secondly, in Model 2 constructed within the framework of the structural equation model, Hypothesis 2 was confirmed. It was determined that the personality structure has an inverse relationship with digital addiction among university students, which suggests that certain personality traits might have a reducing effect on digital addiction. Studies consistently reported that technology-related addictions are closely related to individuals’ personality traits (73–78). For instance, in a study carried out in Korea in 2021 with 400 individuals aged between 20 and 40 years, it was found a significant relationship between personality, mindfulness, stress, and internet gaming addiction tendencies (78). In a study carried out in Malaysia with 301 participants to investigate the relationships between personality traits and Facebook addiction, it was found that personality dimensions were significantly associated with Facebook addiction (79). As indicated in the literature and considering the results achieved in this study, there is a significant relationship between university students’ personality traits and the level of digital addiction. When examining studies that investigate the relationship between digital addiction and personality traits in individuals, it has been found that in the study prepared by Gezer (80) on the explanatory effect of university students’ personality traits on their levels of digital addiction, the personality trait variables together explained approximately 11% of the variance in the digital addiction variable. It has been determined that there is a negative and significant relationship between the levels of openness to experience and the levels of agreeableness among university students’ personality traits and their levels of digital addiction (80). Al-Nawaiseh (81) conducted a study to uncover the degree of digital addiction and personality patterns. According to the study, a significant relationship was found between personality patterns and the degrees of digital addiction among the sample members. It was emphasized that it is important to pay attention to personality structures when creating treatment plans and improving students’ behavior or achievements (81). In their study, Rachubińska et al. (82) stated that there is a relationship between participants’ personality types and internet addiction. As emphasized in the same study, there is a positive relationship between neuroticism and openness to experience and internet addiction, whereas there is a negative relationship between conscientiousness and internet addiction. No significant relationship was found between extraversion and agreeableness and internet addiction (82). In a study conducted by Kızılay (83) with individuals aged 18–55, it was stated that the levels of self-discipline and agreeableness were negatively correlated with the level of internet addiction. According to the results of the study conducted by Kapudere (84), the sub-dimensions of being self-disciplined, being responsible, and experiencing emotional instability are negatively correlated with problematic internet use. In the study conducted by Çınar and Mutlu (85) examining university students’ personality traits, self-esteem, attention, and fear of missing out levels in relation to internet addiction, it was found that there is a positive relationship between attention deficit hyperactivity disorder and neurotic personality traits with internet addiction, while a negative relationship was identified with the responsibility sub-dimension of personality traits. In the meta-analysis conducted by Ji, Yin, Zhang, and Wong (86), it was determined that the only factor strongly associated with Internet gaming disorder was self-control, and it was found to be significantly negatively related to the personality traits of agreeableness and conscientiousness, as well as to the self-related factors of self-resilience and self-efficacy. It is not possible to describe and interpret digital addiction behaviors without considering individuals’ personality traits (87) because personality traits can affect individuals’ behaviors and be associated with various aspects of behavior. Therefore, when investigating the dynamics underlying university students’ digital addiction behaviors, it is essential to prioritize the consideration of personality traits. Additionally, working on individuals’ personality traits may expedite the resolution process of the digital addiction problem by reducing individuals’ addiction levels and tendencies.

(The Big Five, mindfulness, and the relationship with digital addiction).

Thirdly, in Model 3 constructed within the scope of the structural equation model, Hypothesis 3 was confirmed. The predictive relationship of both variables with *Digital Addiction* is indicated to be negative. In this sense, it was determined that specific personality types and levels of conscious mindfulness have a mitigating effect on digital addiction in university students and serve a protective function against this negativity in young individuals. No studies combining these three concepts could be found in the literature review. There are studies examining digital addiction or its subtypes with different variables. In a study carried out by Choi et al. (78) on the mediating effects of personality, mindfulness, and consciousness on the effect of stress on the tendency toward internet gaming addiction, it was found that stress has a positive relationship with the tendency toward internet gaming addiction, and conscientiousness mediates the effect of stress on the tendency toward internet gaming addiction. Considering the results obtained in a study carried out by Kim et al. (39) both young and older adults, it was emphasized that mindless behaviors have negative effects on smartphone usage and health outcomes. It was found in a study carried out by Yang et al.’ (39) that conscious awareness regulates the relationships between mobile phone addiction and both anxiety and depression. As reported in the study carried out by Keskin (88) on the relationship between primary school students’ levels of digital game addiction, psychological resilience levels, and levels of conscious awareness, there is a negative and moderate relationship between digital game addiction and psychological resilience. As can be seen in all these results, individuals’ personality traits and levels of mindfulness have a significant impact on their attitudes toward digital addiction. It can be argued that personality and conscious awareness play a reducing role in digital addiction and serve as a protective function against this negativity in university students. Therefore, the personality traits and levels of conscious mindfulness of university students cannot be ignored in the prevention of digital addiction. Given the excessive use of technology by university students and their vulnerabilities, the results of the current study can contribute to planning awareness-raising activities in the educational environment and working on individuals’ personality traits to prevent digital addiction or other technology-based addiction types.

In conclusion, it can be said that personality structure and the level of conscious awareness have a negative predictive relationship with digital addiction, and that the level of conscious awareness, in addition to personality traits, has a protective and regulatory function against digital addiction in the study group.

As seen in the findings of this study, individuals’ personality traits (the Big Five) and conscious awareness had a strong impact on their levels of digital addiction. Therefore, in studies conducted with students, attention should be paid to the Big Five and conscious awareness.

## 5 Conclusion

Examining the contents of studies, it can be seen that there is no study investigating the relationship between personality, digital addiction, and conscious awareness in university students. The contribution of this article lies in filling a gap in the literature by identifying potential predictors of digital addiction. First of all, it was determined in this study that the prediction between personality and conscious awareness was found to be positive and significant. Accordingly, the personality structure is considered a factor that enhances conscious awareness in university students. Secondly, the prediction between personality and digital addiction was found to be negative and significant. Therefore, certain personality traits, when at a high level, are considered factors reducing digital addiction in university students. In conclusion, it can be stated that the personality structure and the level of conscious awareness negatively predict digital dependency. Besides personality traits, the level of conscious awareness in the study group is suggested to have a protective and regulatory function against digital addiction. Considering that digital addiction is an important problem today in line with the relevant literature, it is evaluated that the findings obtained from the study indicate significant results both theoretically and practically. The finding that personality traits predict digital addiction to a certain extent suggests that individuals may be at risk for digital addiction in terms of certain personality traits. In addition, the level of conscious awareness has also been found to be an important predictor of digital addiction. Therefore, community mental health experts recommend that policymakers in education and social organizations with the potential to reach large audiences develop preventive and interventionist practices related to conscious awareness and digital addiction. Additionally, by identifying individuals’ personal characteristics, it is recommended to strengthen the positions of those prone to psychological issues such as digital addiction through various applications and interventions. The digital environment and tools that cause digital addiction have become an indispensable element in education and all areas of life. In this regard, the conscious and correct use of digital technologies has become an important problem awaiting a solution. According to the findings of this study, personality traits and levels of conscious awareness are significant determinants in students’ use of digital tools. Therefore, when planning preventive interventions for digital addiction, it would be appropriate to prefer educational content that takes into account the personality traits and conscious awareness levels affecting digital addiction behavior.

## 6 Limitations, future studies, and suggestions

The strength of this study is that three variables are examined simultaneously: personality traits, mindfulness, and digital addiction. On the other hand, other studies have focused on the effects of one or two variables. There are several limitations that need to be addressed in future research based on the findings of this study. In this study, the measurement of the personality variable using the Big Five is a limitation. In addition, the analysis of the Big Five total score is another limitation, as the model did not work when the sub-dimensions of the Big Five scale were examined. Another limitation of this study is that the participants were selected using a non-random method, the convenience sampling method. Due to the cost and time constraints and the lack of a sampling frame, the results obtained from the data collected using this method cannot be generalized to the entire population, but they provide a basis for forming an opinion on the subject. Another limitation of the study is that confounding variables were not controlled. The last limitation of the study is that the effect sizes of the predictor variables were not calculated. The results obtained here indicate that the participants had a moderate level of digital addiction and mindfulness. Future studies should investigate these effects on a sample of individuals with a high level of digital addiction. The current study was conducted as a cross-sectional study to test the mediation effect. Future studies should include applied research based on research findings. Although efforts were made to create a homogeneous sample, limitations in terms of generalizability should be taken into account. In addition, the subjective nature of measuring personality, digital addiction, and mindfulness variables is a limitation as participants may have given biased responses. Using an online survey form to administer the surveys is also a limitation, as it is believed that measurement errors can be minimized if the surveys are administered face-to-face. Digital environments and tools that cause digital addiction have become indispensable elements in education and all areas of life. From this perspective, the conscious and correct use of digital technologies has become an important problem to be solved. According to the findings of this study, students’ personality traits and mindfulness levels are important determinants in the use of digital tools. Therefore, when planning preventive interventions for digital addiction, it would be appropriate to prefer educational content that takes into account personality traits and mindfulness that affect digital addictive behavior.

## Data Availability

The datasets presented in this study can be found in online repositories. The names of the repository/repositories and accession number(s) can be found in the article/supplementary material.
